# Could video assisted CPR improve treatment in complex cardiac arrest situations? − A case report

**DOI:** 10.1016/j.resplu.2024.100836

**Published:** 2024-12-13

**Authors:** Steinar Einvik, Ole Erik Ulvin, Trond Nordseth, Oddvar Uleberg

**Affiliations:** a Department of Emergency Medicine and Pre-hospital services, St. Olav s University Hospital, NO-7006, Trondheim, Norway; b Department of Anaesthesia and Intensive Care, St. Olav s University Hospital, NO-7006, Trondheim, Norway; c Department of Research and Development, Norwegian Air Ambulance Foundation, N-0184 Oslo, Norway; d Department of Circulation and Medical Imaging, Norwegian University of Science and Technology, NO-7006 Trondheim, Norway; e Department of Research and Development, Division of Emergencies and Critical Care, Oslo University Hospital, Oslo, Norway

**Keywords:** CPR, CPRIC, Resuscitation consciousness, OHCA, Cardiac arrest, Telemedicine, Emergency medical services

## Abstract

**Background:**

Immediate recognition of cardiac arrest, start of cardiopulmonary resuscitation (CPR) and early defibrillation are key factors to improve survival rates. However, there is considerable variation in the quality of bystander CPR. Video assisted CPR (V-CPR) has been shown to possibly improve CPR quality provided by bystanders. Since 2020, Norwegian emergency medical dispatchers have used V-CPR to increase dispatcher situational awareness and improve on-scene response.

**Case presentation:**

We present a case with witnessed out-of-hospital cardiac arrest (OHCA) in a 58-year-old male with known cardiac disease. Two laypersons present were assisted in CPR with the use of V-CPR. This was complicated by no previous CPR training in both laypersons, long ambulance response times and CPR induced consciousness (CPRIC).

**Conclusions:**

The case represents a complex cardiac arrest with prolonged CPR, CPRIC, two bystanders with no previous CPR training, where V-CPR was instrumental in providing on scene guidance and in decision-making. A more tailored approach to a complex OHCA with long lasting resuscitation was enabled, where high quality CPR was performed and no rescue breaths were given prior to EMS arrival.

## Introduction

Out-of-hospital-cardiac arrest (OHCA) is a significant health concern with approximately 275,000 annual cases in Europe and 420,000 in the United States.^1^ Immediate recognition of cardiac arrest, initiation of cardiopulmonary resuscitation (CPR) and early defibrillation are key factors to improve survival.^2^ However, bystander CPR are only reported on average in 58 % (range 13 % to 83 %) of episodes.^2^ In addition, there is considerable variation in the quality of bystander CPR, with only a minority demonstrating high quality CPR.^3^ Simulation studies have shown that video assisted CPR (V-CPR) may improve cardiac arrest recognition, improve the quality of basic CPR and that it can be performed in a real-life setting.^4^, ^5^ In OHCA, clinical studies show promising results of V-CPR on CPR quality, return-of-spontaneous-circulation (ROSC) rates and survival to hospital discharge.^6^, ^7^, ^8^, ^9^ However, there are considerable knowledge gaps regarding possible benefits of applying this technology in patients with cardiac arrest.^10^ We present a case with witnessed OHCA, where dispatcher V-CPR was initiated early and considered essential to provide a tailored approach to a complex OHCA.

## Case Description

A 58-year-old male with known paroxysmal atrial fibrillation (AF) sustained an OHCA at 9:47 a.m. while changing tires on his jacked-up car together with his son. An emergency call was received at 9:48 a.m. The patient was reported unconscious with agonal respiration and no CPR was performed. Guided by the Norwegian Index for Medical Emergency Assistance, a stepwise tool for decision-making and to guide callers in emergency medical situations by the Emergency Medical Coordination Centre (EMCC), the patient was identified as unresponsive and not breathing normally.^11^ The medical dispatcher immediately instructed the two laypersons on-scene to perform CPR, which they did without hesitation. None had previous CPR experience. Emergency medical services (EMS) and the on-call general practitioner were also immediately dispatched. No other emergency resources (e.g. volunteer first responders) were available during the incident. Since there were two persons on-scene, the medical dispatcher decided to initiate a video call (VC) to better guide the ongoing layperson CPR. The video transmission was initiated two minutes after the emergency call and showed high-quality chest compressions, and a patient with apparent spontaneous respiration and limb movements. After three minutes, they paused compressions according to Norwegian CPR guidelines.^12^ The medical dispatcher then observed that the patient‘s breathing pattern changed into agonal respiration. When compressions were continued, the respiration was again considered normal. Because the breathing pattern was deemed normal due to well performed chest compressions, the medical dispatcher decided not to instruct in mouth-to-mouth ventilations according to standard CPR guidelines. The patient therefore received compression-only CPR until arrival of EMS at 10.21 am, 33 min after start of bystander CPR, at which point V-CPR was discontinued. The two laypersons alternated in performing CPR during the whole incident prior to EMS arrival.

On EMS arrival, the patient was still in cardiac arrest and presented with ventricular fibrillation (VF). A direct current (DC) defibrillation with 200 Joule was delivered by EMS personnel before they continued CPR. The patient obtained ROSC at 10:23 am. However, the ROSC only lasted for 30–40 s, and the patient resumed to VF. Another two DC 200 J defibrillations were delivered, but the patient remained with VF. A physician-staffed air ambulance helicopter arrived on-scene at 10:30 am. With ongoing CPR with the patient in VF, the patient showed signs of life with head and limb movements, verbal sounds and a normal respiration pattern with a respiration rate of 12 breaths per minute during chest-compressions. A fourth DC 200 Joule defibrillation was delivered which resulted in sustained ROSC with AF rhythm at 10:33, 46 min after the time of cardiac arrest. Before transportation a rapid sequence induction with intravenous (iv) analgesia (fentanyl 0.2 mg iv), sedation (ketamine 75 mg iv), muscle relaxation (rocuronium 50 mg iv) and endotracheal intubation was performed without any complications. An arterial line was inserted and an intravenous ketamine infusion (1.5 mg/kg/hour) was started for maintenance of sedation during transport to hospital. The patient was hemodynamically stable in-flight with a blood pressure of 150/80 and a pulse rate of 70–90/min, except a brief period of ventricular tachycardia during take-off from the scene. A dose of intravenous amiodarone 100 mg was administered, and the patient converted to AF. The measured oxygen saturation level was 98 % and the patient was normothermic. Transport to the nearest university hospital was uneventful and he was admitted to hospital at 11:59 a.m.

The first arterial blood gas showed a metabolic acidosis with pH 7.20, pCO2 5.7 kPa, pO2 12.2 kPa, base excess −11.2 mmol/L, lactate 5.5 mmol/L, bicarbonate 17 mmol/L and glucose 13.6 mmol/L. Other values were normal. A percutaneous coronary intervention was performed with a successful revascularisation and stent implementation of partly occluded left anterior descending and circumflex arteries. A one-chamber cardioverter-defibrillator was implanted and he was discharged to a local hospital for follow-up on day 11, with full neurological recovery.

## Discussion

Access to timely EMS response in time-critical situations (e.g. cardiac arrest) is a vital factor to improve patient outcome.^13^ The EMS response time in this case was 33 min, which is often the case in sparsely populated areas, such as rural Norway.^14^ However, the massive development of smartphone technology during the last two decades has opened new telemedicine possibilities in modern healthcare.^15^ The use of VC to assist callers and EMS has the potential to compensate for longer response and travel times. However, despite considerable interest in this technological development, the evidence of effect on patient survival and outcome in real-life OHCA is limited as the specific effect of V-CPR has been difficult to isolate. Other promising technological developments, such as the use of drones carrying defibrillators in OHCA, have shown the potential for increased access to advanced devices and additional decision-making, especially in rural areas.^16^, ^17^ However, like V-CPR, there is a need for further testing in real-life situations to evaluate efficiency and how these interventions impact final outcome.^16^, ^17^ A recent *meta*-analysis found that V-CPR can improve bystander CPR during simulated cases, but that the process is substantially affected by poor video signals and lack of guidance procedures.^18^ Several studies have found improvements in specific items of the CPR process, with better compression rates and hand placement.^10^, ^5^, ^6^, ^7^ In our opinion, the main effect of V-CPR in this case was a more tailored approach and thorough guidance of laypersons without previous CPR experience in a complex setting with long-lasting CPR without back-up of present EMS, resulting in high-quality CPR, CPR induced consciousness (CPRIC) and a good neurological outcome.

VCs were introduced in 2020 in Norwegian EMCCs for the use at the medical dispatcher’s discretion, in order to increase dispatcher situational awareness and improve the quality of care given by lay persons.^19^ Currently, no specific guidelines exist on how VC can be used in the most appropriate way. The solution used in this case was developed by the Norwegian Air Ambulance Foundation and implemented in EMCC nationally in collaboration with the Norwegian Directorate of Health in 2020.^20^ The EMCC sends a link to the caller’s mobile phone after approval by the caller, and the video link allows the medical dispatcher to use the available mobile phone camera.^20^, ^21^ Preliminary evaluations pertaining to general use have described high user-friendliness and that the medical dispatcher’s perception of patient’s acuity was affected in about half of the cases.^19^ We strongly believe that video assistance by trained medical personnel has future unsolved potential. However, it is important to recognize the need for 1) clear guidance protocols 2) training in video-assisted dispatch and 3) training in the ability to instruct lay rescuers in psychological distress. It is also important to keep in mind, that the availability of live video transmission from scenes may expose the EMCC dispatchers to unpleasant visual impressions.^22^ .

Apparent signs of life (i.e. breathing and head/limb movements) were observed in this case during compressions by the laypersons and after arrival of EMS personnel. This situation added extra complexity, which potentially could have affected the decision to continue or stop ongoing CPR. In case reports and a systematic review describing the phenomena, the observation of breathing efforts has been inconsistently reported.^23^, ^24^ Agonal gasps or breathing efforts also may be present in patients with cardiac arrest not receiving CPR, so this sign may be less sensitive on CPRIC than movements. Increasing number of CPRIC events are reported, most likely due to a combined effect of community CPR responder programs, improved CPR quality and increased focus on the chain of survival.^25^ A noteworthy point is that in a prospective study focusing on cognitive experiences in cardiac arrest survivors, only 2 % of patients retrieved visual or auditory awareness following their incident and no patients remembered any experience of pain. ^26^ In the described case, the patient could not recall any memory of the incident.

## Conclusion

The case represents a complex cardiac arrest with long-lasting CPR, signs of life and no previous CPR experience in lay persons, where V-CPR was instrumental in providing on-scene guidance and decision-making.

## CRediT authorship contribution statement

**Steinar Einvik:** Writing – review & editing, Writing – original draft, Investigation, Data curation, Conceptualization. **Ole Erik Ulvin:** Writing – review & editing. **Trond Nordseth:** Writing – review & editing. **Oddvar Uleberg:** Writing – review & editing, Writing – original draft, Supervision, Investigation, Data curation, Conceptualization.

## Declaration of competing interest

The authors declare that they have no known competing financial interests or personal relationships that could have appeared to influence the work reported in this paper.

## References

[1] Go A.S., Mozaffarian D., Roger V.L. (2014). Heart disease and stroke statistics – 2014 update: a report from the American Heart Association. Circulation.

[2] Perkins G.D., Graesner J.T., Semeraro F. (2021). European resuscitation council guidelines 2021: executive summary. Resuscitation.

[3] Chocron R., Jobe J., Guan S. (2021). Bystander cardiopulmonary resuscitation quality: potential for improvements in cardiac arrest resuscitation. J Am Heart Assoc..

[4] Ecker H., Lindacher F., Adams N. (2020). Video-assisted cardiopulmonary resuscitation via smartphone improves quality of resuscitation: a randomised controlled simulation trial. Eur J Anaesthesiol..

[5] Ecker H., Wingen S., Hamacher S., Lindacher F., Boettigger B.W., Wetsch W.A. (2021). Evaluation of CPR quality via smartphone with a video livestream - a study in a metropolitan area. Prehosp Emerg Care..

[6] Linderoth G., Rosenkrantz O., Lippert F. (2021). Live Video from bystanders' smartphones to improve cardiopulmonary resuscitation. Resuscitation.

[7] Bielski K., Bottiger B.W., Pruc M. (2022). Outcomes of audio-instructed and video-instructed dispatcher-assisted cardiopulmonary resuscitation: a systematic review and meta-analysis. Ann Med..

[8] Lee H.S., You K., Jeon J.P., Kim C., Kim S. (2021). The effect of video-instructed versus audio-instructed dispatcher-assisted cardiopulmonary resuscitation on patient outcomes following out of hospital cardiac arrest in Seoul. Sci Rep..

[9] Lee S.Y., Song K.J., Shin S.D., Hong K.J., Kim T.H. (2020). Comparison of the effects of audio-instructed and video-instructed dispatcher-assisted cardiopulmonary resuscitation on resuscitation outcomes after out-of-hospital cardiac arrest. Resuscitation.

[10] Sykora R., Peran D., Renza M., Bradna J., Smetana J., Duska F. (2022). Video emergency calls in medical dispatching: a scoping review. Prehosp Disaster Med..

[11] National Competance Center for Prehospital Emergency Medicine. Norsk indeks for medisinsk nødhjelp (NIMN) (Norwegian Index for Medical Emergency Assistance) 4th edition. 2018.

[12] Norwegian Resuscitation Council. 2021 Guidelines for adult basic life support. https://nrr.org/images/pdf/2023/Norwegian_2021_basic_life_support_guidelines-English%20summary_published280423.pdf. Accessed 19 October 2024.

[13] Wilde E.T. (2013). Do emergency medical system response times matter for health outcomes?. Health Econ..

[14] Kjærvoll H.K., Andersson L.J., Bakkelund K.E., Harring A.K.V., Tjelmeland I.B.M. (2023). Description of the prehospital emergency healthcare system in Norway. Resusc plus..

[15] Bhavnani S.P., Narula J., Sengupta P.P. (2016). Mobile technology and the digitization of healthcare. Eur Heart J..

[16] Lim J.C.L., Loh N., Lam H.H. (2022). The role of drones in out-of-hospital cardiac arrest: a scoping review. J Clin Med..

[17] Kristiansson M., Hagiwara M.A., Svensson L. (2024). Drones can be used to provide dispatch centres with on-site photos before arrival of EMS in time critical incidents. Resuscitation.

[18] Pan D.F., Li Z.J., Ji X.Z., Yang L.T., Liang P.F. (2022). Video-assisted bystander cardiopulmonary resuscitation improves the quality of chest compressions during simulated cardiac arrests: a systemic review and meta-analysis. World J Clin Cases..

[19] Kramer-Johansen J, Brattebo G, Zakariassen E, Riddervold I, Hjortdahl M, Idland S, et al. Evaluation report regarding video use in Norwegian emergency communication centres. 2021. https://kudos.dfo.no/documents/48330/files/30756.pdf. [In Norwegian] Accessed 29 November 2024.

[20] The Norwegian Air Ambulance Foundation. The video solution «Hjelp 113 Video». 2023. https://norskluftambulanse.no/eng/the-video-solution-hjelp-113-video/ Accessed 29 November 2024.

[21] The Norwegian Air Ambulance Foundation. Video used in emergency calls over 150,000 times in Norway. 2023. https://norskluftambulanse.no/eng/video-used-in-emergency-calls-over-150000-times-in-norway/ Accessed 29 November 2024.

[22] Idland S., Iversen E., Brattebø G., Kramer-Johansen J., Hjortdahl M. (2022). From hearing to seeing: medical dispatchers' experience with use of video streaming in medical emergency calls - a qualitative study. BMJ Open.

[23] Olaussen A., Shepherd M., Nehme Z., Smith K., Bernard S., Mitra B. (2015). Return of consciousness during ongoing cardiopulmonary resuscitation: a systematic review. Resuscitation.

[24] Pound J., Verbeek P.R., Cheskes S. (2017). CPR induced consciousness during out-of-hospital cardiac arrest: a case report on an emerging phenomenon. Prehosp Emerg Care..

[25] Howard J, Lipscombe C, Beovich B, Shepherd M, Grusd E, Nudell NG, et al. Pre-hospital guidelines for CPR-Induced Consciousness (CPRIC): A scoping review. Resusc Plus. 2022:12:100335. 10.1016/j.resplu.2022.100335. eCollection 2022 Dec. 10.1016/j.resplu.2022.100335 PMC9713363 36465817

[26] Parnia S., Spearpoint K., de Vos G. (2014). AWARE—AWAreness during REsuscitation—a prospective study. Resuscitation.

